# The role of DNA sequence in nucleosome breathing

**DOI:** 10.1140/epje/i2017-11596-2

**Published:** 2017-11-30

**Authors:** Jamie Culkin, Lennart de Bruin, Marco Tompitak, Rob Phillips, Helmut Schiessel

**Affiliations:** 1 Institute Lorentz for Theoretical Physics, Leiden University, Niels Bohrweg 2, 2333 CA Leiden, The Netherlands; 2 Laboratory for Computation and Visualization in Mathematics and Mechanics, École polytechnique fédérale de Lausanne, Lausanne, Switzerland; 3 Department of Applied Physics and Division of Biology and Biological Engineering, California Institute of Technology, Pasadena, CA 91125, USA

## Abstract

Roughly 3/4 of human genomes are sequestered by nucleosomes, DNA spools with a protein core, dictating a broad range of biological processes, ranging from gene regulation, recombination, and replication, to chromosome condensation. Nucleosomes are dynamical structures and temporarily expose wrapped DNA through spontaneous unspooling from either end, a process called site exposure or nucleosome breathing. Here we ask how this process is influenced by the mechanical properties of the wrapped DNA, which is known to depend on the underlying base pair sequence. Using a coarse-grained nucleosome model we calculate the accessibility profiles for site exposure. We find that the process is very sensitive to sequence effects, so that evolution could potentially tune the accessibility of nucleosomal DNA and would only need a small number of mutations to do so.

## 1 Introduction

Eukaryotic DNA is highly compacted within chromatin to fit inside the nucleus of the cell. For humans, for example, 46 DNA molecules of a total length of about two meters must be made to fit in a micrometer-sized nucleus. The lowest level of its organisation, the nucleosome, has been resolved to atomic resolution by X-ray crystallography [1,2]. A nucleosome consists of 147 base pairs (bp) of DNA (about one persistence length) wrapped around a protein cylinder composed of eight histone proteins. Histone-DNA interactions mainly involve hydrogen bonding between the negatively charged DNA phosphates and positively charged elements at the surface of the octamer, and are localized at 14 distinct binding sites where the minor groove of the DNA faces the octamer. This forces the DNA into a super-helical path of $1\frac{3}{4}$ turns.

Having detailed knowledge of the nucleosome structure, together with an increasing theoretical understanding of sequence-dependent DNA elasticity, makes it possible to build detailed nucleosome models. Such models, as well as increasingly sophisticated experiments, have started to bring to light the important role of DNA sequence for the conformational and dynamical properties of nucleosomes, as highlighted in a recent review [3]. A picture emerges of the nucleosome as being not one specific DNA-protein complex but rather a whole class of complexes, with significant variation in their physical properties.

This idea has mainly been studied in the context of the sequence-dependent affinity of DNA to the histone octamer, which contributes to the positioning of nucleosomes along genomes (alongside other effects, such as competition for DNA binding with other proteins, and the active positioning by chromatin remodelers [4]). This is sometimes referred to as the “nucleosome positioning code” [5] (see also refs. [6] and [7] for earlier versions of this idea). The degeneracy of the genetic code allows for these mechanical cues to be written even on top of genes [8]. Concrete examples where mechanical cues play a role *in vivo* are nucleosome depleted regions before transcription start sites in unicellular organisms, which facilitate transcription initiation [9,10]; mechanically encoded retention of a small fraction of nucleosomes in human sperm cells, allowing for transmission of paternal epigenetic information [11]; and the positioning of 40% of all nucleosomes around nucleosome inhibiting barriers in human somatic cells, whose function has yet to be determined [12].

However, the role of DNA mechanics goes beyond determining nucleosome affinity. A recent experiment [13] studied the response of three different nucleosomes (different with respect to their bp sequence) to an external force. Even though their sequences are closely related, these three nucleosomes unwrap under force either from the left end, from the right end, or symmetrically, and this can be understood from the mechanical properties of the wrapped sequences [14]. This finding is also consistent with an experiment where DNA bound to a nucleosome is mechanically unzipped providing a histone-DNA interaction map [15]; the nucleosome contains the same sequence, 601, as used for one of the nucleosomes in ref. [13] and shows the same asymmetry, when comparing forward and backward unzipping traces. It has been shown that such asymmetric nucleosomes present “polar barriers” for RNA polymerases making transcription in one direction more efficient than in the other [16], see also [15].

Nucleosomes also unwrap from the protein cylinder simply due to thermal fluctuations. This process, called “nucleosome breathing” or “site exposure”, is widely studied for its potential role in essential DNA processes like gene regulation. For example, it is known that DNA is inaccessible to proteins when it is bound to histones [3]. Breathing may be crucial in providing the required access *in vivo*.

The first experiments to probe nucleosome breathing measured the accessibility of enzymatic restriction sites *in vitro* throughout a wrapped nucleosomal sequence [17,18]. The authors found that the accessibility of different sites within the nucleosome decreased exponentially as a function of how deeply buried they are within the nucleosome. The two studies reported significantly different accessibility profiles, reflecting the fact that each study used a different wrapping sequence. One study employed a natural positioning sequence [17], the other an even stronger artificial positioning sequence [18].

However, interpreting the differences in the results of the two studies has proven challenging. The artificial sequence is known to wrap the nucleosome more tightly than the natural sequence and, as expected, appears to be less accessible in the nucleosome breathing experiment. However, by the same reasoning, the accessibility of the artificial sequence would be expected to decay more quickly towards the dyad, which is not observed. A later study on the natural sequence [19] also seems to generate results inconsistent with the earlier experiment [17]. We will attempt to understand these discrepancies, using a bp-level model of the nucleosome.

Our nucleosome model, which we have tested extensively [8,10,14,20,21], reveals the sensitivity of site exposure to sequence variation. For experiments that modify the DNA sequence as part of the experimental methodology, as is for instance done in [18], this sensitivity has the potential to distort the results. In the big picture, this sensitivity also suggests that genomes may easily have evolved to tune the accessibility of their nucleosomal DNA.

Much research on nucleosome breathing has been performed since refs. [17,18] were published, mainly based on FRET, which has enabled more direct probing of the dynamics of site exposure [22–43] (for a review, see also [44]). However, the interpretation of data gathered through this technique has proved less straightforward [45] than that of the older experiments employing restriction enzymes [17–19,46–48]. Therefore, we restrict ourselves in this study to comparison with the older results. Moreover, various computational models for site exposure exist [45,49–56] but cannot be used for the current study because they do not account for sequence effects. There are two exceptions: the first exception is ref. [57] that presents a theoretical breathing profile for the 601 nucleosome based on an all-atom molecular dynamics simulation; we will discuss this model in sect. 3.1. The second exception is ref. [58] which, however, is not based on a microscopic model but is trained on high-throughput maps of nucleosome positions.

In the next section we will introduce our model of the nucleosome, which allows us to predict the sequence-dependent accessibility along the nucleosomal DNA. In sect. 3 we present the accessibility profile for the sequence of ref. [18] and discuss how the inhomogeneous mechanical properties of the sequence manifest themselves in the accessibility. To better understand these effects, we will look at idealized sequences. In sect. 4 we will take a closer look at the two restriction enzyme experiments [17,18] and compare the experimental findings to the predictions of our computational nucleosome model. Finally, we present our conclusions in sect. 5.

## 2 Methods

### 2.1 Nucleosome model

We employ the same nucleosome model as in our previous work [8,14,20,21]. The DNA is represented by the rigid bp model (RBP) [59] which treats each bp as a rigid plate, the spatial position and orientation of which are described by six (three translational and three rotational) degrees of freedom. It assumes only nearest-neighbor interactions with a quadratic deformation energy between successive bp [59]:

(1) $$E=\frac{1}{2}\left(q-q_{0}\right)\cdot K\cdot \left(q-q_{0}\right).$$

Here *q* is a six-component vector that describes the relative degrees of freedom between two base pairs. The intrinsic, preferred values of these degrees of freedom are given by *q*_0_. The properties of the (six-dimensional) springs connecting the base pairs are given by *K*, a six-by-six stiffness matrix. The sequence dependence of the model comes into play because the stiffness (*K*) and intrinsic shape (*q*_0_) of a given bp step depend on its chemical identity. These parameters can be found in the literature [59,60], and we use the same hybrid parameterization [61] as in [8,14,20,21].

**Fig. 1.**
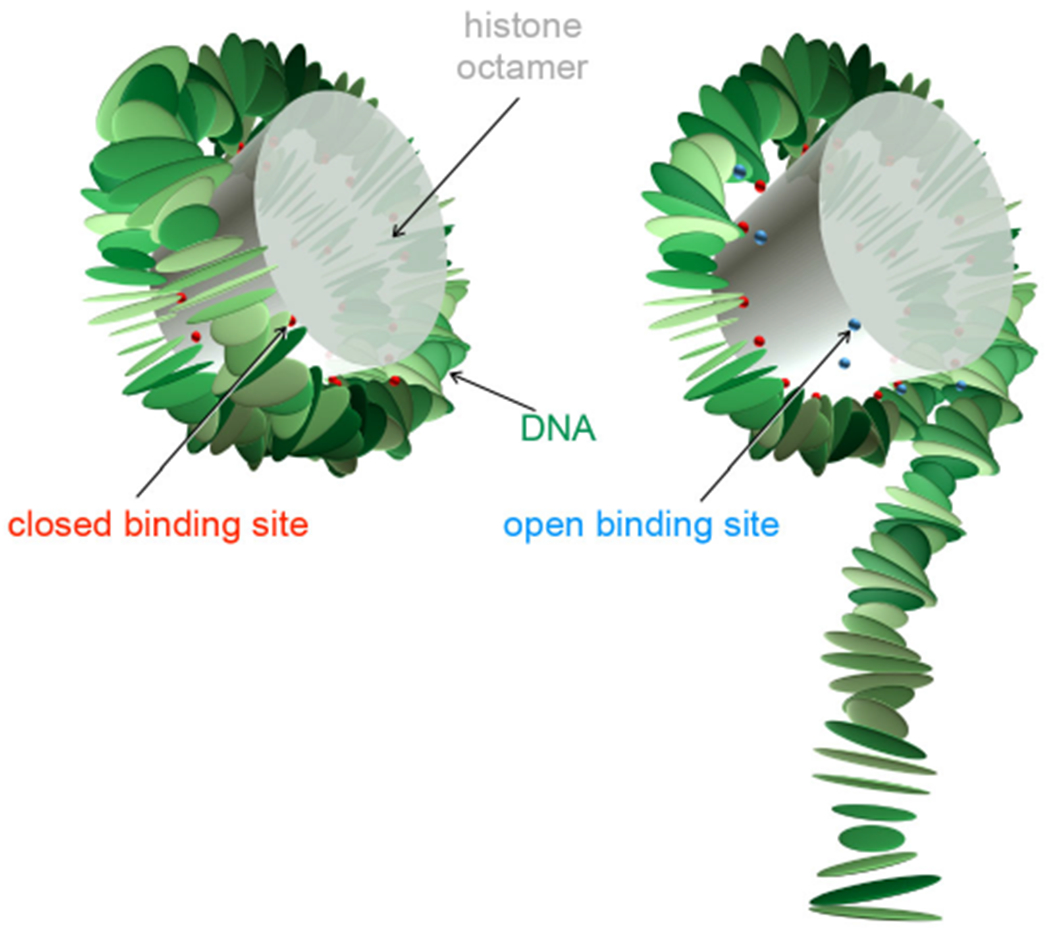
Nucleosome model with the fully wrapped complex on the left and a partially unwrapped complex on the right. Each rigid plate represents a bp, the locations of the constraints (corresponding to bound phosphates) are shown by beads, two per binding site. Red beads represent closed sites and lue beads open sites. The cylinder is a rough representation of the protein core but is not simulated explicitly (except through the binding sites).

The DNA is forced into a super-helix through a set of 28 constraints that represent the 14 binding sites to the histone octamer (see fig. 1 (left)) and which were extracted from the nucleosome crystal structure without introducing free parameters [8]. These constraints correspond to bound phosphates in the DNA backbone. In the context of the RBP model, these bound phosphates are accounted for through fixed midplanes for the bp steps involved (see ref. [8] for details). The positions of these constraints are listed in table 1.

We allow the binding sites of the nucleosome to be opened at the expense of some adsorption energy, leading to partially unwrapped states as depicted in fig. 1 (right). We know from mechanical DNA unzipping experiments [15] that different binding sites have different adsorption strengths and we have incorporated this effect in earlier work [14,20,62]. However, the differences in the binding strength can only be estimated roughly and we found the experimental data to be too noisy to be able to detect such nuances. Therefore, for the current study we assume equal binding strength for all sites. This reduces the adsorption energy to a single free parameter.

The nucleosome model itself has been extensively tested. We have shown in ref. [8] that our model reproduces the nucleosome positioning rules, gives good estimates of relative affinities between various sequences and predicts the rotational positioning of nucleosomes. In ref. [14] it reproduced details of the sequence-dependent response of nucleosomes to tension recently reported in ref. [13]. We have also used an approximation to this model [63] to perform genome-wide analyses of the nucleosome affinity of promoter regions [10] and found it to be in good agreement with nucleosome maps in various organisms.

For a given sequence we calculate the energy landscape of the nucleosome as a function of unwrapping as follows. For each possible state of unwrapping, we produce random samples of nucleosome conformations using the standard Metropolis algorithm, and calculate the average energy. Each Monte Carlo move consists of a local conformational move of a randomly picked base pair. These moves are chosen such that constraints corresponding to fixed binding sites are not violated (as detailed in ref. [8]).

### 2.2 Calculating accessibilities

**Fig. 2.**
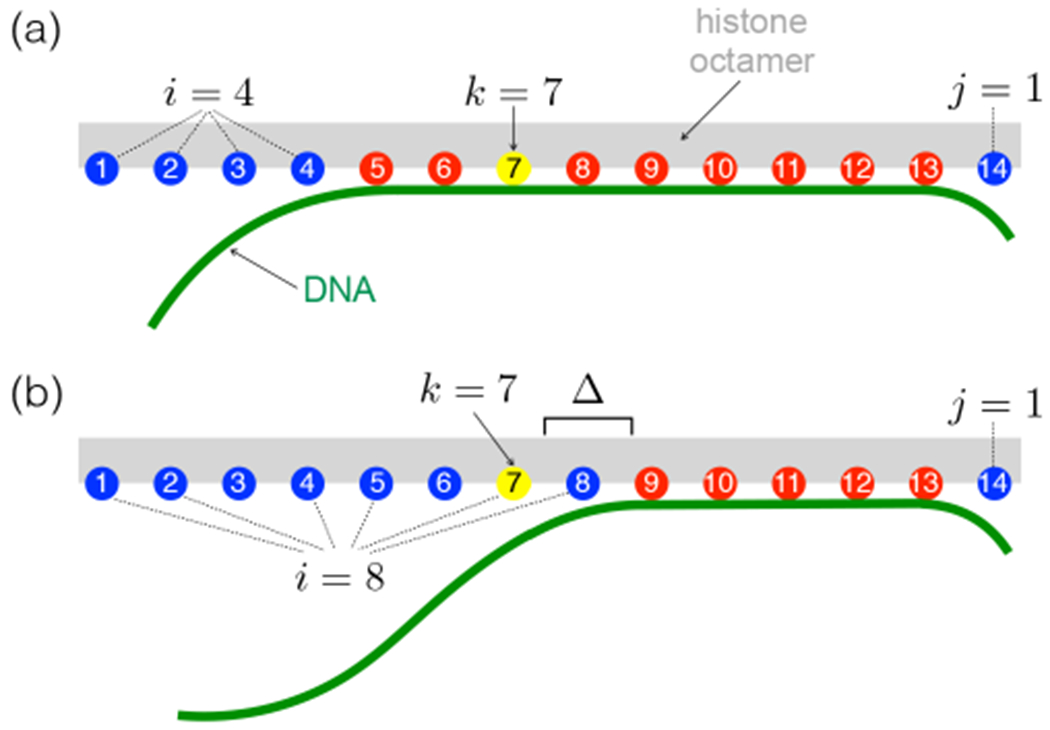
Schematic depiction of unwrapping states where site *k* (yellow) is (a) inaccessible and (b) accessible. The number of sites opened from the left and right are given by *i* and *j*, respectively. *Δ* is the number of additional sites that need to be opened beyond a site to make it accessible. Same colour code as in fig. 1.

We label the set of possible nucleosome configurations as (*i, j*) with *i* binding sites unbound from the left and *j* from the right (see fig. 2), and with at least one site still bound (the fully unwrapped state has negligible probability, because it costs adsorption energy, while no elastic energy is gained). To unbind a site, we remove the two corresponding constraints on the relevant phosphates.

The experiments we examine rely on restriction enzyme binding to the nucleosomal DNA. Due to their finite size, the enzymes may require additional DNA to be unbound to allow access [51]. In principle, the different sizes and geometries of the enzymes may affect the additional exposure required. For simplicity, when computing an accessibility profile, we assume that all enzymes require the same amount of additional DNA to be unbound. We account for this by introducing an additional free parameter *Δ*, which we call the steric accessibility, *i.e.* the number of additional binding sites that need to be opened to allow enzyme access.

The overall probability *P_k_* that a site *k* is accessible to an enzyme (the equilibrium constant for site exposure) is then the sum of the probabilities of all configurations in which it is accessible (and at least one site is still bound). Figures 2(a) and (b) show states with site *k* inaccessible and accessible, respectively. Stated in two terms corresponding to opening site *k* through unwrapping from the left and from the right, respectively (see also ref. [51]):

(2) $$P_{k}=\frac{1}{Z}\left(\sum_{i\geq k+\Delta ,i+j<14}C_{ij}+\sum_{j>14-k+\Delta ,i+j<14}C_{ij}\right).$$

Here *C_ij_* is related to the effective adsorption energy *E_ij_*, *i.e.* the total pure adsorption energy plus the total elastic energy of the nucleosome in state (*i, j*), by

(3) $$C_{ij}=\exp\left(-\frac{E_{ij}}{k_{B}T}\right),$$

and *Z* is the partition function of the system, the sum over *C_ij_* for all configurations, in which at least one site remains bound:

(4) $$Z=\sum_{i+j<14}C_{ij}.$$

The model has two free parameters: the (pure) adsorption energy per site, *E*_ads_ (*i.e.* state (*i, j*) has a total adsorption energy (14 − *i* − *j*) × *E*_ads_), and the number of extra unbound sites required, *Δ*, see fig. 2(b). In appendix A we outline approximations to this model in which smaller sets of configurations (*i, j*) are considered to enable more efficient computation; because the accessibility of the DNA decreases exponentially towards the dyad, most of the configurations make negligible contributions to the accessibility of a given site in eq. (2).

## 3 Analysis of idealised breathing profiles

In sect. 4 we will fit the model to experimental data, but here we first illustrate the dependence of the breathing profiles on the effective adsorption energy (the adsorption energy taking into account the sequence-dependent cost of deforming the DNA) and steric accessibility.

### 3.1 601 and ideal breathing profiles

We first consider the widely studied 601 sequence [64], and compare it to that of an “ideal” sequence for which the elastic energy is homogeneously distributed along the nucleosome. This will allow us to illustrate the effect of inhomogeneous sequence effects, such as we find for the 601 sequence.

In order to compare the 601 sequence to the idealized nucleosome, we require a reasonable estimate of *E_ads_*. For the nucleosome to remain stably wrapped, but still be able to breathe, it has been argued [51] that the average adsorption energy should be greater in magnitude than the elastic energy by ~ 1–2 *k_B_T* per binding site. Therefore, in the following we choose the effective adsorption energy in this range. The precise value is not important here since this energy just affects the steepness of the breathing profile (as we show below) but does not affect other features.

**Fig. 3.**
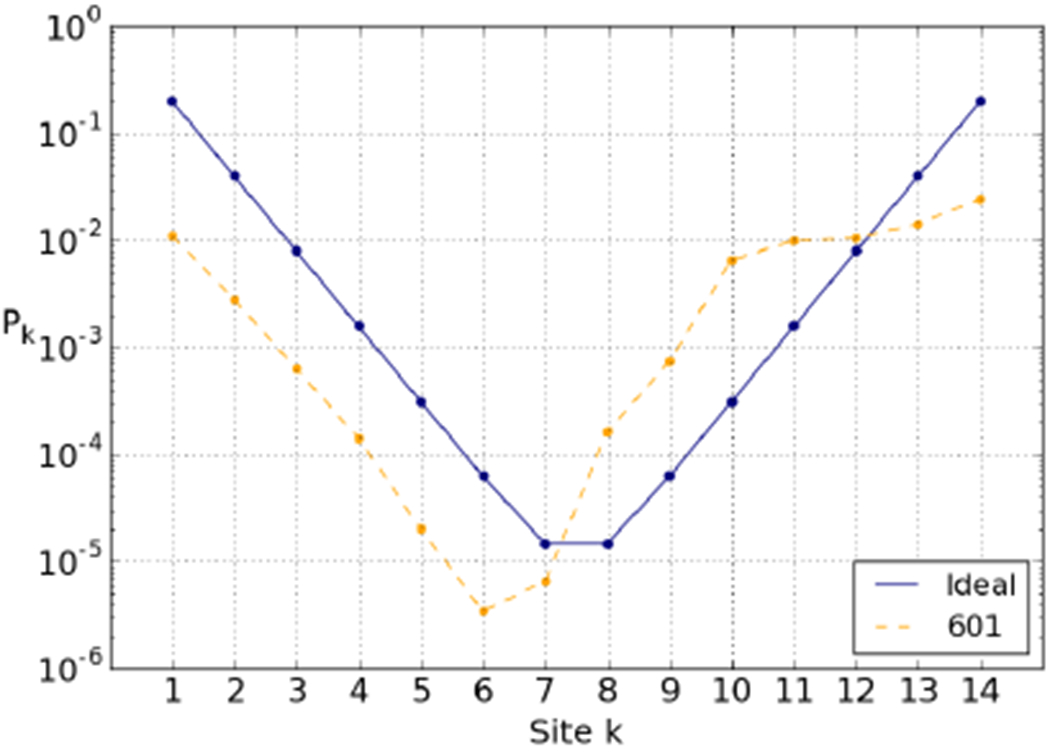
601 and ideal breathing profiles calculated using eq. (2). *P_k_* is the equilibrium constant for site exposure, the fraction of time the *k*-th position is open (and thus accessible to, *e.g.*, a restriction enzyme) in equilibrium conditions. In each case, the total elastic energy is 65.5 *k_B_T* and the total adsorption energy is chosen to be −91 *k_B_T*. The steric accessibility is set here to *Δ* = 0. Note the logarithmic scale.

For the “ideal” sequence this cost is exactly the same for each site opening, leading to an exponential decay of the Boltzmann weight with the number of bound sites. In the leading-term approximation (appendix A), the accessibility of the left-hand sites (*k* = 1, …, 7) can be given as

(5) $$P_{k}\approx \frac{C_{k,0}}{C_{0,0}}=\frac{\exp\left(-E_{k,0}/k_{B}T\right)}{\exp\left(-E_{0,0}/k_{B}T\right)}\;=\exp\;\left(-\left(\frac{E_{0,0}}{k_{B}T}+kS\right)+\frac{E_{0,0}}{k_{B}T}\right)=\text{exp}\left(-kS\right),$$

where *S* is the effective cost to open a binding site (*i.e.* the energy change associated with breaking the site, *E*_ads_, and straightening the freed DNA stretch) in units of *k_B_T*. Similarly, for the right-hand side, *P_k_* ≈ exp(−(15 – *k*)*S*), see fig. 3.

The 601 breathing profile (also depicted in fig. 3) also shows exponential decay towards the dyad, but in comparison with the “ideal” profile there are a few notable features: a clear asymmetry, in that the right side is much more accessible than the left side; a shift of the location of the least accessible site(s); and the entire left-hand side is shifted downward. All these features are due to the unequally distributed elastic energy, which leads to different effective costs to open sites. Compared to the 601 profile provided in fig. 5C in ref. [57] (based on an all-atom simulation) our profile is more asymmetric. It shares an easily accessible outer stretch at the right end but does not show such a symmetrically related stretch at the left end. The reason why the two models differ in their prediction for this end is not clear at this point. The experimental data [18] feature a similar asymmetry as our model but a direct comparion is not useful as there are various complications to be accounted for, as explained later in this paper.

**Fig. 4.**
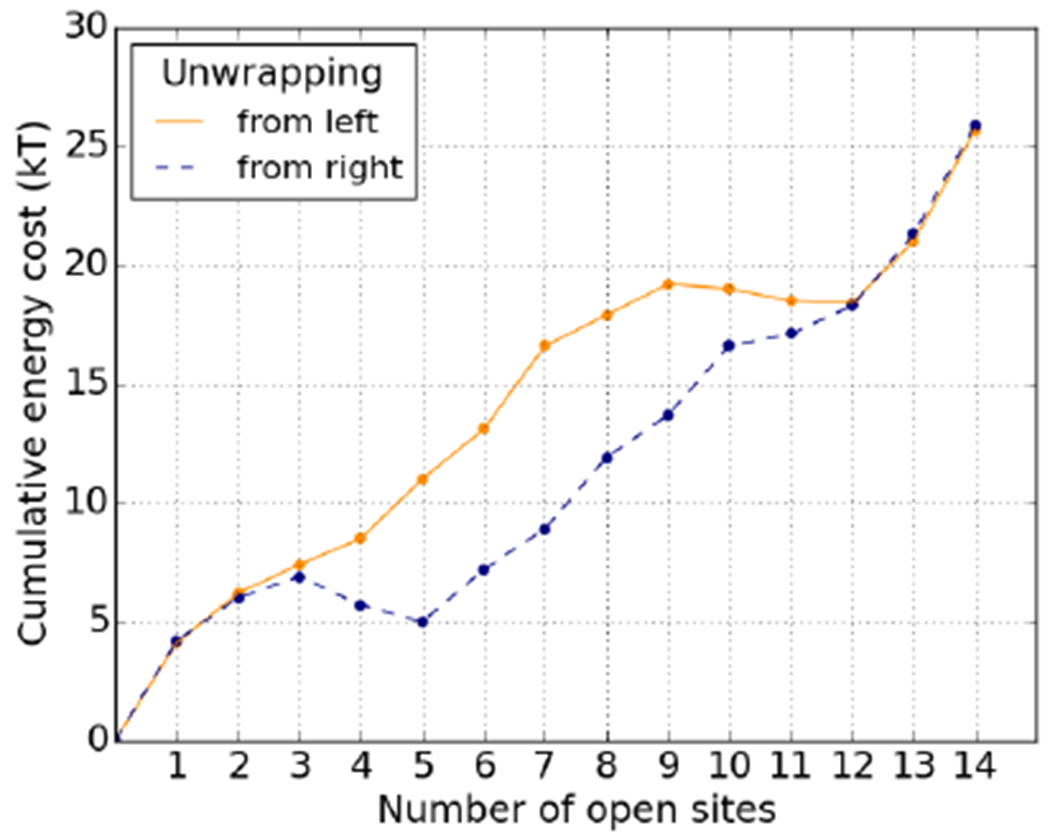
Cumulative energy cost of unpeeling the 601 sequence from either the left or right end. Note that the cumulative cost is not strictly monotonic. There are sections of the DNA where the elastic energy gained by unwrapping is larger than the cost in adsorption energy.

The 601 sequence is known to unwrap asymmetrically under force, preferentially from its right-hand side, and it has been suggested that this is due to that half of the DNA being stiffer, *i.e.* that it stores more elastic energy [13,14]. Our predicted elastic energies agree: the third quarter is the most expensive to deform, and the right-hand side is overall stiffer than the left-hand side. As shown in fig. 3, the breathing profile exhibits the expected asymmetry. In fact, we find that the third quarter has a higher elastic energy than adsorption energy. This means that unbinding sites in the third quarter is energetically favourable—these are “free” sites, in the sense that it costs nothing to unbind them. This is illustrated in fig. 4, in which the cumulative energy cost of unwrapping from either side is plotted. Unwrapping from the right, we see that going from state (0, 1) to state (0, 5) adds very little to the cumulative cost, leading to the increased accessibility of the right-hand side.

### 3.2 Effect of the effective energy profile on breathing

In the previous section we saw how the inhomogeneous distribution of the elastic energy found in the 601 nucleosome strongly affects the accessibility profile. In this section we will take a closer look at how alterations to the effective adsorption energies of the binding sites affect the accessibility. We will use *Δ* = 0 throughout, and address non-zero values in the next section.

**Fig. 5.**
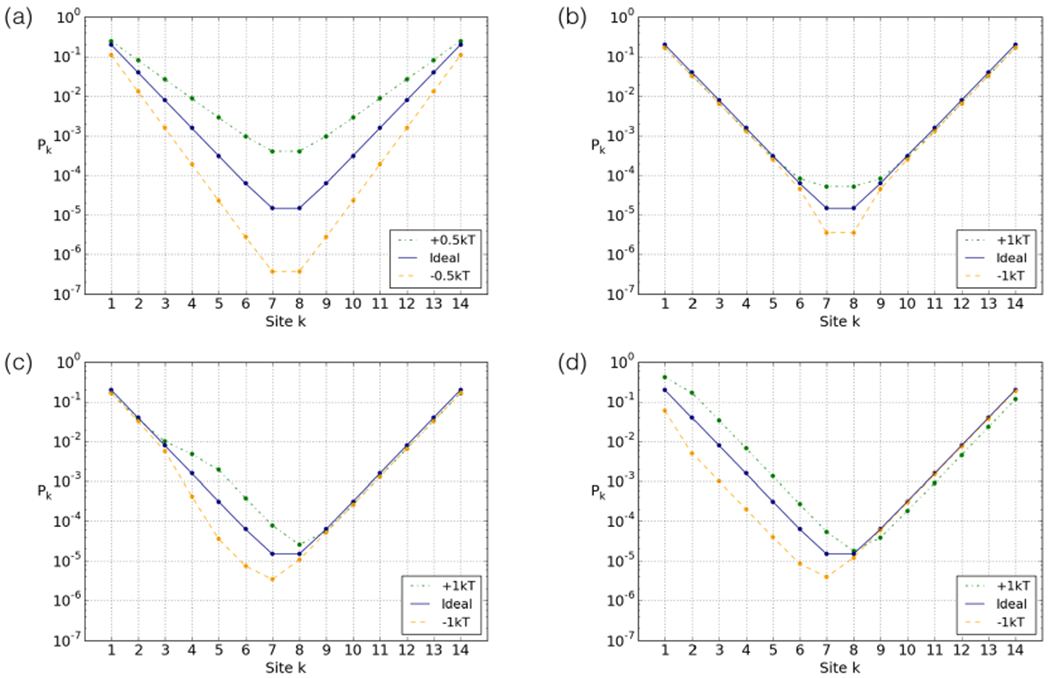
Ideal breathing profiles resulting from alterations to the effective adsorption energy distribution for: (a) all sites, (b) sites 7 and 8, (c) sites 4 and 5 and (d) sites 1 and 2. The ideal sequence fully wrapped has 65.5 *k^B^T* elastic and −91 *k_B_T* adsorption energies, so −25.5 *k_B_T* effective adsorption energy.

Figure 5(a) shows that an increase in effective adsorption energy applied uniformly across the nucleosome decreases the accessibility of all sites. Moving inward towards the dyad, the effects are stronger, because the inner sites require the outer sites to be opened first. We can interpret the results easily if we apply the leading-term approximation from appendix A (eq. (A.3)). Shifting the effective adsorption energy of each binding site by *ψ*, we can approximate

(6) $$P_{k}\approx e^{-k\left(S+\psi \right)}.$$

Thus we find a simple modification to the exponential decay.

The shape of the profile is significantly altered by unevenly distributed energy. Changes to the effective adsorption energies of a site causes a cascading effect towards the dyad, as shown in fig. 5(b)–(d).

The approximation from appendix A again enables a straightforward understanding of this effect: if we shift the energy of a binding site that lies between a given site and the nearest end from which it is accessed by *ψ*, roughly a factor of exp(−*ψ*) is applied to its accessibility.

This cascading effect can also be clearly detected in the 601 breathing profile (fig. 3). Due to the relatively small amount of elastic energy stored around the two leftmost sites, the effective cost of opening these sites is high, impeding access. This propagates down the left-hand side, shifting the overall accessibility down (similar to fig. 5(d)), and shifting the minimum to the left.

### 3.3 Effect of steric accessibility on breathing

In sect. 2 we defined the free parameter *Δ* in the breathing model: the number of open adjacent sites required for enzymatic access. A theoretical study [51] has argued that 30 bp must be unbound either side of a site for it to be accessible, which corresponds roughly to *Δ* = 3, since there are ~ 10 bp bound between binding sites. We will fit this parameter to the experimental data in sect. 4, but first we examine its impact upon the ideal and 601 breathing profiles.

**Fig. 6.**
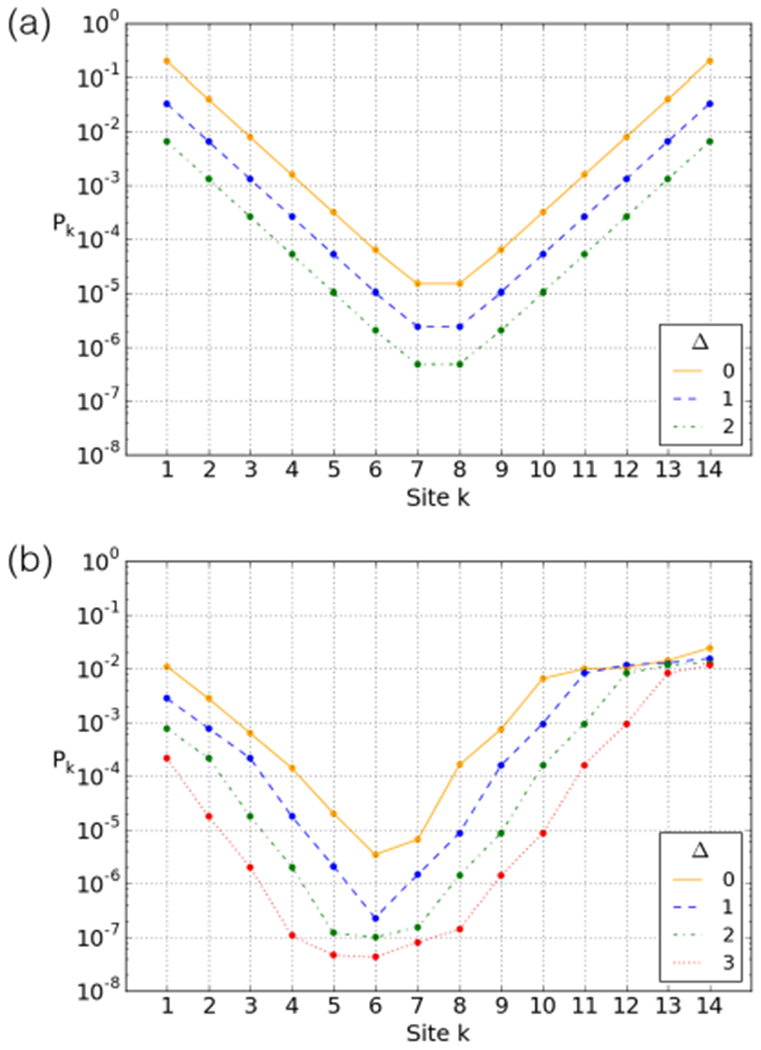
Breathing profile for the (a) “ideal” and (b) 601 sequences at different values of *Δ*, the extra number of open sites required either side for an enzyme to bind to a site.

Figure 6(a) shows the effect of *Δ* for the “ideal” sequence. As can be seen, increasing *Δ* decreases all site accessibilities by a constant factor. This is captured by the leading-term approximation simply by realising that the first configuration that reveals site *k* (*e.g.*, from the left) is configuration (*k* + *Δ*, 0), which results in

(7) $$P_{k}\left(\Delta \right)\approx e^{-\left(k+\Delta \right)S},$$

*i.e.*, a factor exp(−*ΔS*) is applied to the entire profile. The approximation breaks down for high values of *Δ* (for the ideal profile, *Δ* ≥ 7), at which point the central sites (7 and 8) can only be accessed by complete dissociation of the DNA. Higher values of *Δ* extend this to further adjacent sites (*e.g.*, 6 and 9). However, such high values are not expected.

The effect of *Δ* on the accessibility of the 601 nucleosome is shown in fig. 6(b). The inhomogeneity makes the effect of increasing *Δ* less straightforward. First, the flatness of the accessibility profile at the rightmost binding sites (due to the “free” binding sites discussed in sect. 3.1) is lost for larger values of *Δ*. Second, at *Δ* ≥ 2 a flattening occurs around the minimum of the accessibility profile, widening at higher *Δ*.

The first effect is straightforward: as *Δ* increases, the “free” binding sites can only be accessed by also unbinding the more costly sites further inward. The second effect is actually also due to the “free” sites. At higher *Δ*, the binding sites in the flattening area require these “free” sites to be opened to be accessible, but this costs no additional energy. For instance, for *Δ* = 3, sites 6–8 require unbinding up to sites 9–11, respectively. The latter are all “free” sites, so opening up sites 6–8 comes at no extra cost compared to opening site 5.

## 4 Comparison to experiment

By incorporating restriction sites in a nucleosomal DNA sequence, and exposing the nucleosome to the appropriate restriction enzymes, the transient exposure of specific portions can be measured by counting the number of enzymatically produced DNA fragments [17,18]. The authors used sequences known to have high affinities for the octamer, in order to ensure a well-positioned nucleosome, which is crucial for mapping accessibilities. In the first study [17], a nucleosomal DNA sequence from the sea urchin 5S RNA gene was used, and in the second study [18], the artificial 601 sequence, the sequence with the greatest affinity for the octamer known at the time [64]. In each case the authors inserted restriction sites, creating derivative sequences —5Sa, 5Sb, 5Sc and 601.2— and measured for each restriction enzyme the exposure probability of the corresponding target sites.

**Fig. 7.**
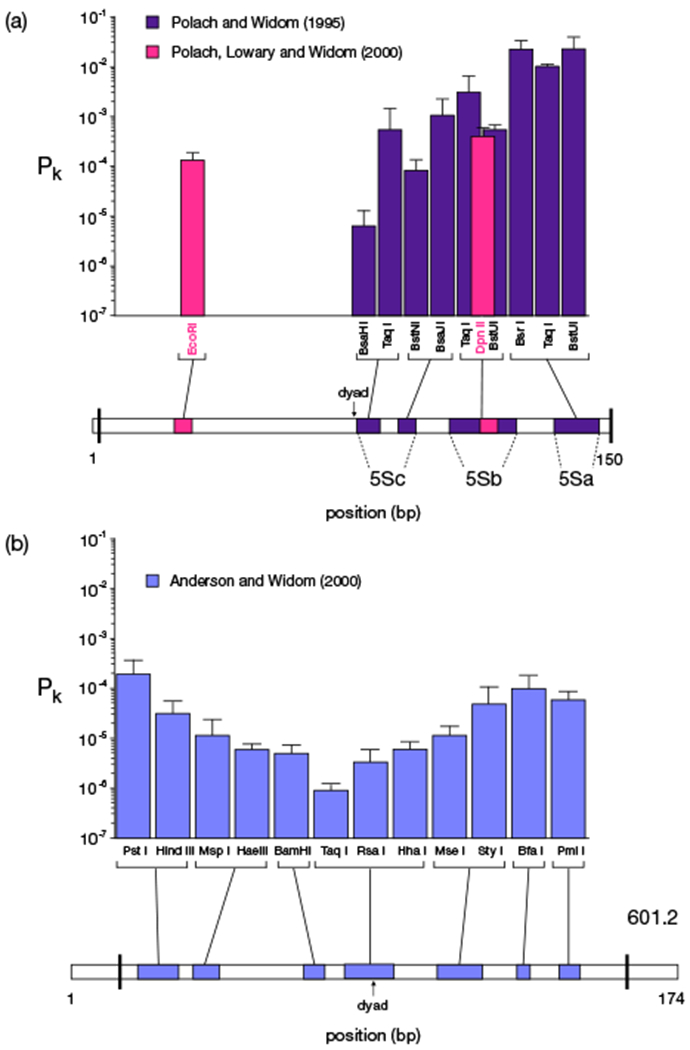
Results of restriction enzyme experiments on nucleosome breathing, carried out by the Widom lab [17–19]. Accessibility profiles for (a) the 5S nucleosome [17,19] and (b) the 601 nucleosome [18]. Below the plots are schematic depictions of the DNA molecules used in the experiments. Oloured stretches indicate the positions of the restriction sites and the black vertical bars indicate the edges of the regions occupied by the nucleosome. Note the non-monotonic decay in accessibility, which is most likely due to the use of different restriction enzymes (which have different shapes and sizes and require different salt concentrations and temperatures) and the varying orientations of the restriction sites with respect to the octamer.

The results from these two experiments are summarised in fig. 7. Both experiments found an exponential decay in accessibility toward the dyad; even the inner-most sites on the wrapped DNA are accessible. Importantly, the breathing profiles for the two sequences are different: the 601 sequence is less accessible overall than the 5S sequence. As the 601 DNA has a higher affinity to the octamer than the 5S DNA, this seems logical. However, since the average site opening cost for the 601 sequence is higher than for the 5S sequence, we expect (see sect. 3.2 and fig. 5(a)) a faster decay. Surprisingly, however, the 5S decay (slope of log(*P_k_*) *versus* binding site number (1.00±0.19 (95% confidence interval))) is steeper than the 601 decay (0.53±0.20 assuming dyad position 88, 0.57 ± 0.34 assuming position 94, see 4.2). Figure 7 suggests that the lower accessibility of the 601 nucleosome is caused not by higher effective adsorption energies, but by an overall shift of the profile, *i.e.* a different value for *Δ.*

Several limitations of the technique, pointed out by the authors [17], must be kept in mind. The steric accessibility *Δ* depends on the orientation (with respect to the octamer) of the binding site (which varies by design in the experimental setups [17,18]) and the size and shape of the enzyme. The equilibrium accessibility depends on the temperature: Polach and Widom [17] present in their fig. 5 the temperature dependence of the equilibrium constants and found large variations, but no systematic dependences, suggesting substantial experimental errors (data presented were averages over all measured temperatures). Moreover, experiments for different restriction enzymes were performed under different ionic conditions, which is known to affect the affinity of DNA to the histone core.

The results of the two restriction enzyme studies are reported per enzyme, and are schematically mapped to the nucleosomal DNA sequences in fig. 7. For each enzyme, we have identified which of the 14 DNA-protein binding sites provide access to the enzyme’s restriction site. For example, the restriction site for *Bsa*HI in 5Sc is at positions 75–80, which are only accessible when site 8 is released (table 1). With this approach, some restriction sites are predicted to be equally accessible, as they are exposed by the same site. This may not be accurate, due to additional hydrogen bonds and for steric reasons, which we cannot account for. Additionally, not all restriction sites can be mapped to a single site, because some lie between two binding sites. An example is *Bsr*I in 5Sa at positions 132–137. It is accessible after either 13 sites from the left, or one site from the right (table 1). In such cases, the closest site is used. Due to the large variance of the data, these nuances do not significantly impact our results.

We have two free parameters in our model: *E*_ads_, the (pure) adsorption strength per site, and *Δ*, the steric accessibility. These are determined by least-squares fitting to the logarithm of the measured accessibility profiles.

### 4.1 The experiments of Polach and Widom (1995)

**Fig. 8.**
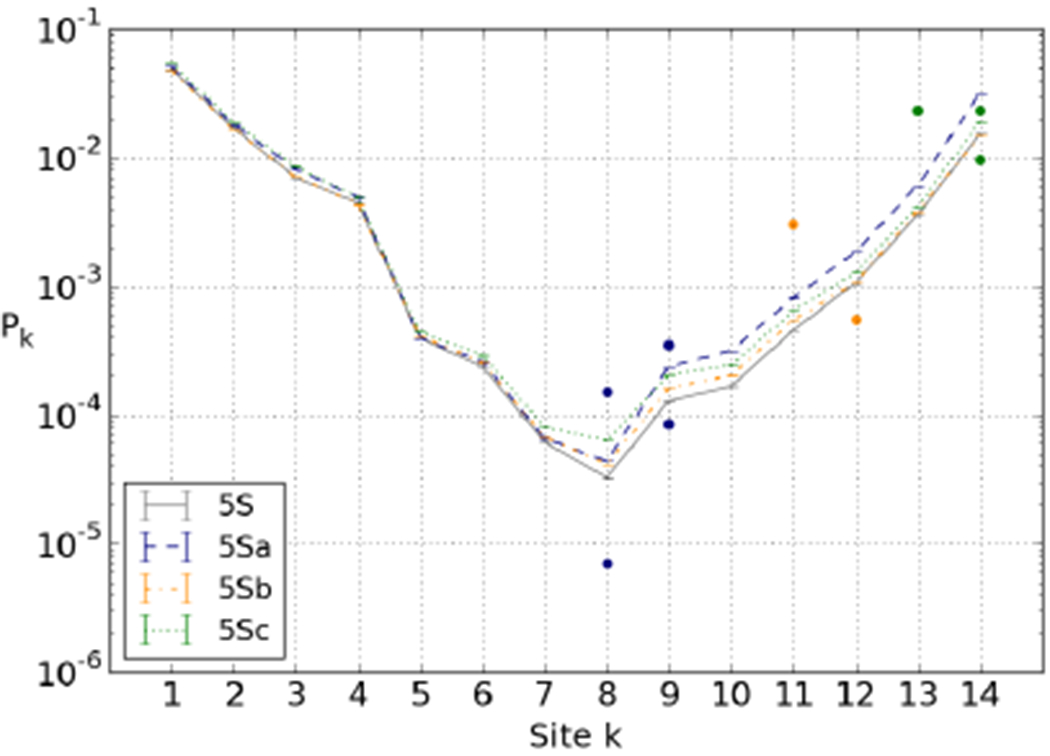
Equilibrium constant for site exposure for the 5S sequence and the three constructs 5Sa, 5Sb and 5Sc used in ref. [17], experimental data (dots) and theoretical fitted predictions (lines). Fitted values of free parameters: adsorption strength per site of 6.3 *k_B_T*, and *Δ* = 0.

Polach and Widom [17] used a 150 bp part of the full 256-bp 5S sequence [65], from which they created three derivative sequences: 5Sa-c, see fig. 7(a). The three sequences together contain 34 modified basepairs, for 9 restriction sites on one half of the nucleosome. An important question is whether the accessibility profiles are affected by these modifications. We calculated the breathing profiles, and found that the elastic energies for the fully wrapped constructs are all < 1 *k_B_T* higher than for the original 5S sequence, in agreement with experimental results (see table 1 in ref. [18]). The alterations do cause some small changes to the right-hand side (where the alterations took place) of the breathing profiles, see fig. 8. We accounted for this by simultaneously fitting our predicted accessibility profiles for the three sequences to the appropriate subsets of experimental data. The best overall fit results in an adsorption strength per site of 6.3 *k^B^T* and *Δ* = 0, and is shown in fig. 8.

### 4.2 The experiments of Anderson and Widom (2000)

In the second study [18] Anderson and Widom altered 15 bp of the 601 sequence, creating 12 restriction sites across almost the entire sequence. Crucially, we believe the authors incorrectly mapped the position at which the nucleosome sits along this new sequence (601.2), potentially due to the then-unknown asymmetric nature of the 601 nucleosome. This impacts the mapping of the restriction sites to the nucleosome and the subsequent breathing profile.

The authors mapped the position of the nucleosome using the enzyme exonuclease III, which digests DNA, removing nucleotides in a stepwise fashion. They assumed that the nucleosomal boundary would cause a long pause in digestion, until the DNA breathing allowed the enzyme to progress. They derived the pauses in digestion from the populations of different lengths of undigested DNA over time, measured using polyacrylamide gel electrophoresis (PAGE) (see fig. 1C, [18]). From the first long pause in digestion from each end, they inferred the nucleosomal boundaries, and placed the dyad between bps 87 and 88 (in the following called the dyad-88 position). However, since this study, it has become known that the dyad for the original 601 sequence is positioned at bp 94 (dyad-94 position) (see, *e.g.*, ref. [13]). This raises the question whether the sequence changes between 601 and 601.2 result in a ~ 6 bp shift of the nucleosome position, or the 601.2 position was misreported.

We found the given PAGE data (only digestion from the left-hand side) to be consistent with the dyad-94 position, once one accounts for asymmetric breathing. There is a very short pause in digestion before the long pause, which the authors did not comment on. However, a short pause is expected because the right-hand side opens so easily.

**Fig. 9.**
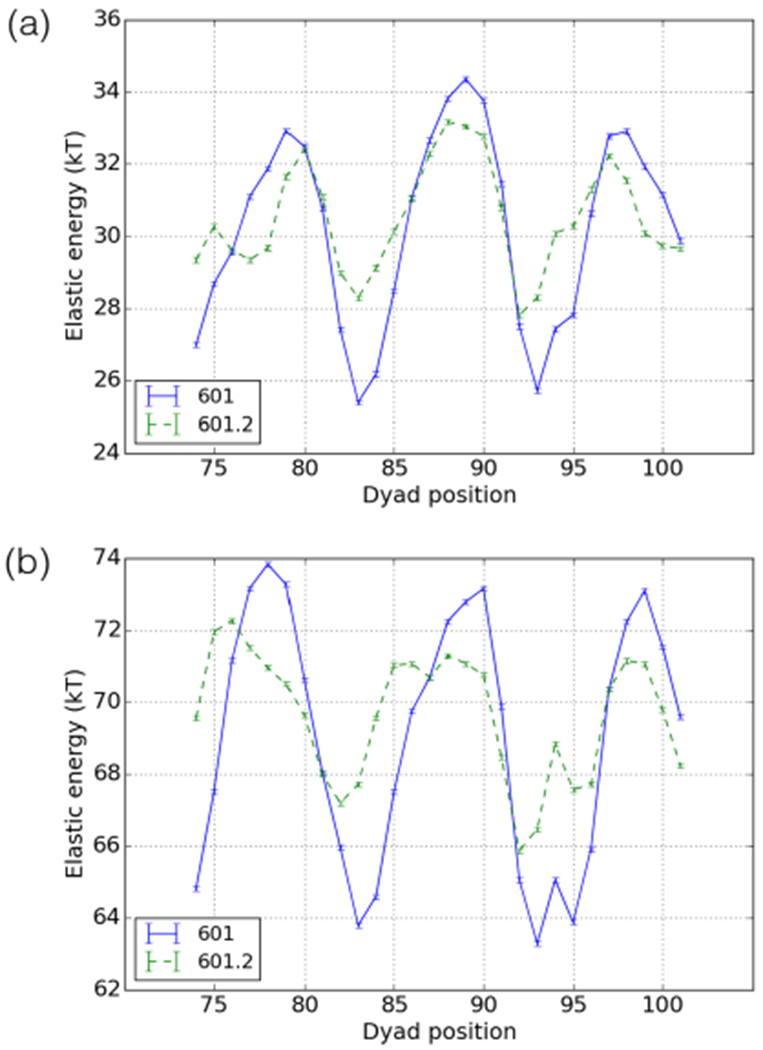
(a) The tetramer energy landscapes for the 601 and 601.2 sequences. Stable positions are predicted at the minima. (b) Same as (a) but for the octamer, *i.e.* the full nucleosome.

To address this, we calculated the nucleosome and tetramer energy landscapes for the 601 and 601.2 sequences (the tetramer assembles onto the DNA first during nucleosome reconstitution and is therefore assumed to strongly influence the preferred position of the nucleosome [5]). These energy landscapes show the effective adsorption energy of the nucleosome as a function of dyad position, the minima being the most stable positions. As shown in the supplemental material of [8], the minima are reliable predictors of nucleosome position; 60% of experimentally mapped nucleosomes on the first yeast chromosome fall within ±1 bp of a minimum. We can see in fig. 9 that the dyad-94 position is very close to minima for both the 601 and 601.2 nucleosome and tetramer energy landscapes. Conversely, all landscapes have a maximum near the dyad-88 position, predicting it to be an unstable position.

**Fig. 10.**
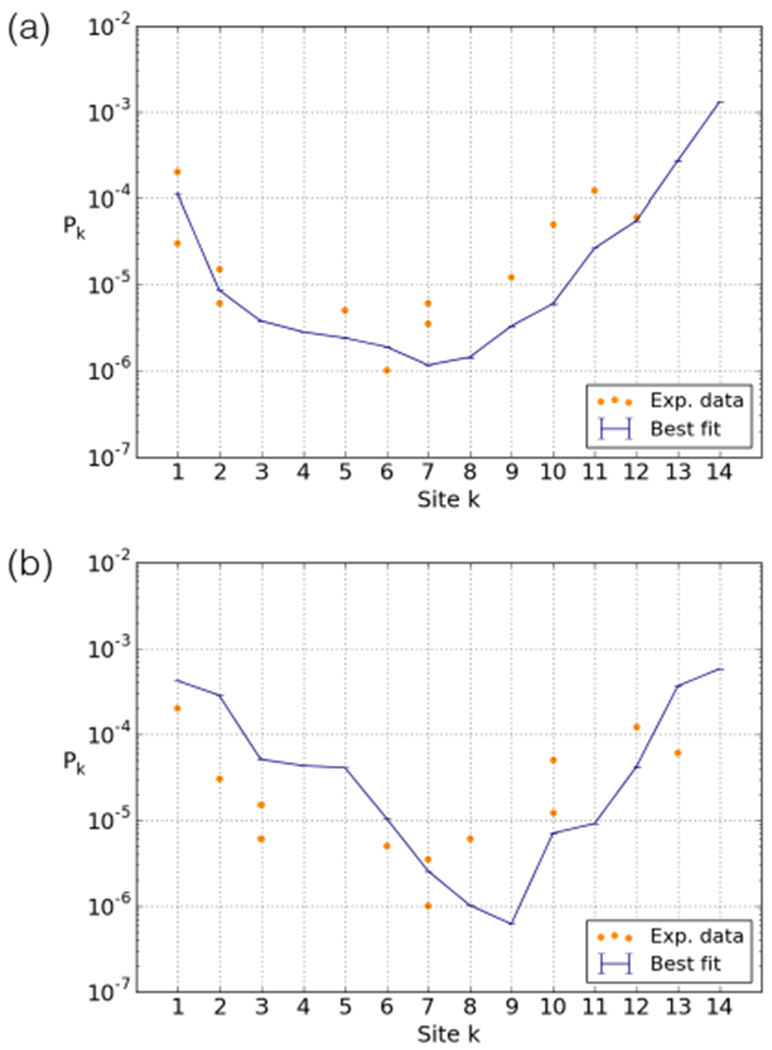
601.2 breathing profiles, showing the best fit of the model on the experimental data for the two nucleosome positions (a) dyad-94 position (6.4 *k_B_T* per site and *Δ* = 5) and (b) dyad-88 position (6.7 *k_B_T* per site, *Δ* = 4).

We mapped the restriction site accessibilities in fig. 7(b) according to the two reported dyad positions, and fit our model to each case. The best fit assuming the dyad-94 position is slightly better, fig. 10(a), than the dyad-88 position, fig. 10(b), reducing the sum of squared residuals by a factor 2.4. Each of the fits results in an energy adsorption per site very similar to 5S (6.4 and 6.7 *k_B_T* as compared to 6.3 for 5S). On the other hand we found unexpectedly high values of *Δ* = 5 and 4 (as compared to 0 for 5S) reflecting the overall shift in the profile. However, we note that this parameter is not very strongly constrained by the data: a fit of similar quality can be achieved for the dyad-88 position using *Δ* = 2.

Based on the available evidence we conclude that the 601.2 position was indeed misreported: the energy landscapes predict the dyad-94 position as stable, and the dyad-88 position as unstable; the fit assuming the dyad-94 position is slightly better; and the PAGE data seems to support either position.

**Fig. 11.**
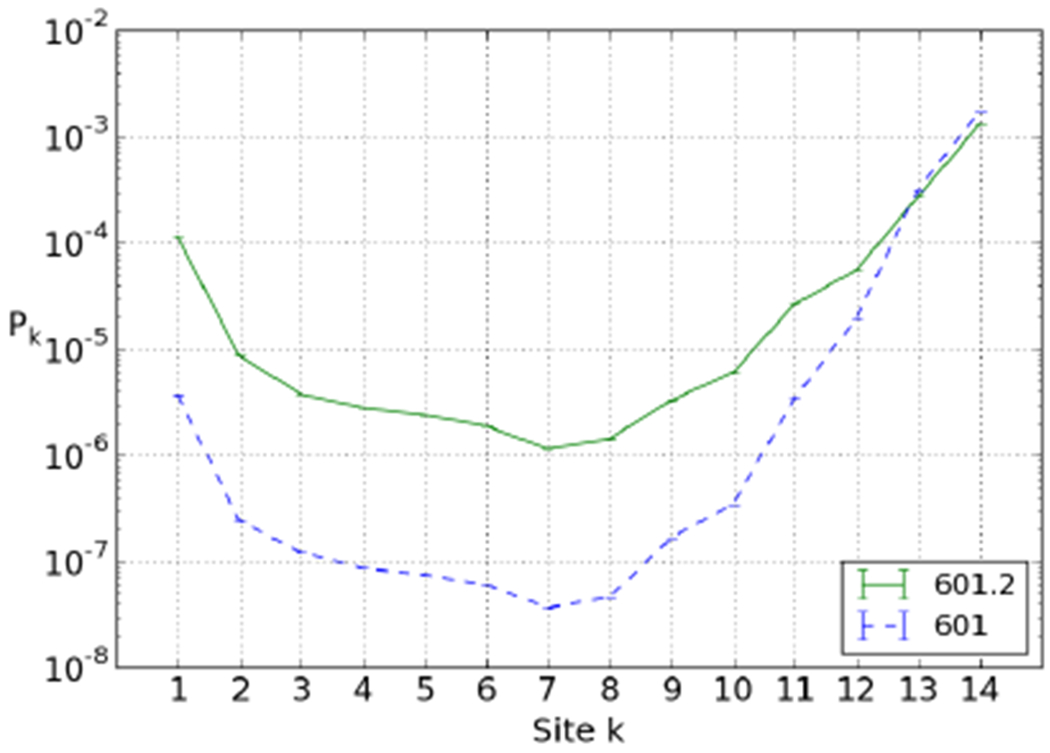
601.2 and 601 breathing profiles compared, assuming dyad-94 nucleosome position. Calculated according to best-fit parameters on 601.2 data: *E*_ads_ = 6.4 *k_B_T* and *Δ* = 5.

To see the effect of the sequence changes between 601 and 601.2, we compared their breathing profiles using the best fit parameters of the 601.2 dyad-94 position. The elastic energy of 601.2 fully wrapped is ~ 3.5 *k_B_T* higher than for 601 (see fig. 9(b)). Experimental results indicate a change in relative affinity of about 3 *k_B_T* (table 1 in ref. [18]). Figure 11 shows that our model predicts a large difference in the two profiles: 601.2 is roughly an order of magnitude more accessible than 601, and shows a significantly less steep decline in accessibility from the right. As outlined in sect. 3.2 and illustrated in fig. 5(a), this is due to the higher average cost to open sites: 1.7 *k_B_T* for the 601 sequence compared to 1.2 *k_B_T* for 601.2. The change in accessibility going from 601 to 601.2 is thus substantial (unlike for the 5S variants) and provides at least a partial explanation for the surprisingly slow decay of the accessibility.

## 5 Conclusions

Using a nucleosome model with sequence-dependent DNA elasticity, we studied the effect of the base pair sequence on the accessibility profile of the nucleosome. Such a computational study allows us to understand in detail how physical properties of the nucleosome emerge from the underlying DNA nanomechanics. We have specifically discussed this in the context of the highly asymmetric accessibility of the 601 nucleosome, see figs. 3 and 4.

The main motivation of this study was to get a better handle on the available experimental data and the apparent discrepancies therein. Unfortunately the data shows large non-systematic variations in the accessibility profiles (*e.g.*, with temperature or between nearby positions) that point to substantial experimental errors. However, our simulations shed some light on the overall differences between the breathing profiles of the nucleosomes derived from the 5S gene [17] (fig. 7(a)) and the 601 sequence [18] (fig. 7(b)).

Two features of the data, the faster decay in accessibility toward the 5S dyad and the overall shift in the 601 accessibility to lower values, are puzzling. The higher affinity of the 601 nucleosome is thought to come about by an overall lower elastic energy of its DNA compared to 5S, distributed all along its wrapped length, especially including the inner half in contact with the tetramer, as the 601 sequence has been selected for high tetramer affinity. The 601 nucleosome should thus show a steeper slope toward the dyad as compared to 5S. But according to the data the low accessibility to the 601 sequence comes about instead through an extremely high cost to reach the outmost stretches of the wrapped DNA that has to overcompensate for a nucleosome that is everywhere else less stable.

Our simulations gave us two important hints. First, it is crucial to realise that the sequences used in the two sets of experiments are not the 5S and 601 sequence themselves but sequences derived from them, each containing several restriction sites. We found these modifications to have a small effect on the accessibility of 5S (fig. 8), but a large effect on 601 (fig. 11). We found that the latter effect partially explains the surprisingly slow decay found in the 601 experiment.

Second, in ref. [18] the position of the 601.2 nucleosome was reported at a position that is 5 bp away from the now-known preferred 601 position. Our analysis suggests that the preferred positions are the same for 601 and 601.2 and that the position reported in ref. [18] is likely due to an erroneous assumption made by the authors.

What our model has not been able to explain is the substantial shift of the accessibility to smaller values going from 5S to 601. We can accommodate this in our fit by using a different steric factor, namely *Δ* = 5 instead of *Δ* = 0. Both values are surprising: *Δ* = 0 suggests that for the 5S nucleosome restriction enzymes gain access to their target site if the binding sites have just opened up to those sites. However, there seems not to be enough room for restriction enzymes to bind as the octamer surface is nearby. Likewise the value of 5 for *Δ* suggests that an extra 50 bp need to be unwrapped from the 601 nucleosome before a restriction enzyme gains access to its target site which seems excessive. It is thus more likely that our fitted *Δ* is capturing other discrepancies between the experiments, or systematic errors in the data.

Based on what we have learned from our model, we see at this point no obvious explanation for this difference. However, in a later paper [19] by the same group on the 5S nucleosome, new values were reported for some of the accessibilities of the outer portion ~ 10^−4^ (see their table 2 and magenta bars in our fig. 7(a)). These values were obtained after correcting for an initial “burst” in the enzymatic digestion, of unknown origin, and they are substantially lower than in the original experiment, and more similar to the 601.2 accessibilities. Lower outer values would require a higher value for *Δ* in the best fit, more in line with the 601.2 sequence. However, there is not enough data to conclude that this explains the shift.

The strong effect we see on the breathing profile due to the modifications to the 601 sequence shows that the breathing behavior of the nucleosome can be altered with relatively few modifications to the sequence. This leads to two conclusions. First, experiments in which sequences are modified, for example to include restriction sites, should be carefully interpreted. The changes to the sequence may lead to unexpected results. Second, the ease with which the behavior is altered means that evolution may also have tailored genomic nucleosomal sequences to exhibit certain breathing properties, if some evolutionary benefit can be obtained from doing so.

We thus see that the sequence-dependent mechanical properties of the DNA double helix not only affect the rotational [8] and translational positioning [10] of nucleosomes, but also other mechanical properties, *e.g.* response to force [14,20] and breathing (current study). It will be interesting to see what other special nucleosomes can be created by tailoring the DNA sequence, and whether real genomes have evolved to encode for such nucleosomes.

More immediately, we hope that our insights will inspire new experimental inquiry into nucleosome breathing, taking into account the various concerns we have pointed out, namely: 1) Nucleosome breathing is very sensitive to sequence changes. 2) Different restriction enzymes work at different temperatures and salt concentrations which affects nucleosome dynamics. 3) Steric effects (enzyme size and rotational positioning of the restriction site with respect to octamer) affect the accessibility of a given restriction enzyme to its target site. Based on this we recommend that the insertion of restriction sites be limited to one such site at a time, to minimize the effects of the mutations on the mechanical properties of the molecules. This also makes it possible to use a single restriction enzyme, which should reduce the variance in the required steric accessibility. For the same reason, we recommend that the orientation of the restriction sites with respect to the octamer be kept homogeneous.

## Figures and Tables

**Fig. 12.**
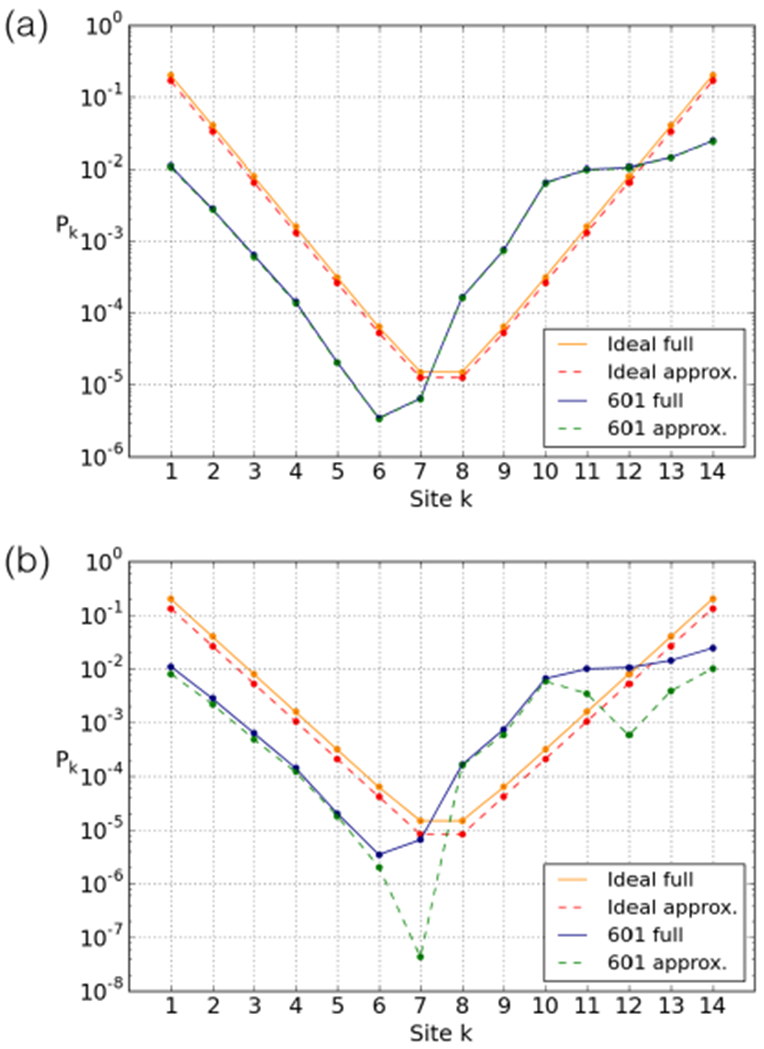
Equilibrium constants for site exposure for the ideal and 601 sequences. Comparison between the full calculation and two approximations: (a) one-arm unwrapping approximation and (b) the leading-term approximation.

**Table 1.** Locations of the bound phosphates of the 14 sites in the nucleosome, as derived from crystallographic data [8]. The phosphates connecting the 147 base pairs are numbered 1, … , 146.

Site	Bond locations (phosphates)
1	3, 7
2	15, 18
3	25, 30
4	35, 39
5	46, 50
6	56, 60
7	66, 70
8	77, 81
9	87, 91
10	97, 101
11	108, 112
12	117, 122
13	129, 132
14	140, 144

## Appendix A. Approximations to calculate accessibilities

There are 105 unwrapping configurations (*i, j*) with one site still bound, and it is computationally expensive to calculate their Boltzmann weights. However, as the energetic cost increases approximately linearly as sites are unwrapped, the Boltzmann probability decays exponentially, and eq. (2) is dominated by a small number of terms. Here we outline two approximations used in this paper.

### One-arm unwrapping approximation

The first approximation we consider is to count only the 26 configurations in which one or the other arm is unwrapped at a time, *i.e.* keeping *i* = 0 fixed whilst *j* > 0 and vice versa. This approximation works well because it is generally significantly more likely that a site is accessed from the end nearest to it, as this requires the fewest binding sites to be opened. In this approximation, the partition function is

(A.1) $$Z\prime =\sum_{i=0}^{13}\exp\;\left(-\frac{E_{i0}}{k_{B}T}\right)+\sum_{j=1}^{13}\exp\;\left(-\frac{E_{0j}}{k_{B}T}\right),$$

and the probability of a site being accessible is

(A.2) $$P_{k}\approx \frac{1}{Z\prime }\left(\sum_{i\geq k+\Delta }C_{i0}+\sum_{j>14-k-\Delta }C_{0j}\right).$$

Figure 12(a) shows that this approximation returns almost exactly the same breathing profiles for both the ideal and 601 sequences, slightly underestimating both. In the ideal profile, the points are shifted ~ 20%. Relative to the variation of the accessibility profile over several orders of magnitude, this error is negligible. The error for 601 is actually even smaller (< 5%). We use this approximation to calculate the figures in sect. 4 of the paper.

### Leading-term approximation

A further approximation is to only take the leading term in calculating the site accessibility; this is useful as it leads to simple approximate expressions for accessibilities which can guide understanding, and is used in sect. 3. Since the probabilities of configurations decrease exponentially with the number of unbound sites, *P_k_* is usually dominated by a single configuration, namely the one with fewest unbound sites. Under this assumption, site accessibility for the left-hand sites is approximated by

(A.3) $$P_{k}\approx \frac{C_{k,0}}{Z\prime \prime },$$

and for the right-hand sites by

(A.4) $$P_{k}\approx \frac{C_{0,15-k}}{Z\prime \prime }.$$

The partition function is approximated by the probability of the fully wrapped configuration

(A.5) $$Z\prime \prime =C_{0,0}=e^{-\frac{E_{0,0}}{k_{B}T}.}$$

Figure 12(b) shows that, using this leading-term approximation, the ideal profile is slightly underestimated (error again < 20%). The 601 profile is not well approximated at certain binding sites. In these cases, either the dominant term is due to unwrapping from the opposite side (site 7), or there is no dominant term —the “free” sites 10 to 12 distort the right-hand side as explained in sect. 3.1. This approximation is therefore useful to guide understanding when considering isolated effects, but cannot be used to predict the breathing profile of a strongly inhomogeneous sequence.

The approximation can be improved by including additional terms. For example, if the energies of the outermost binding sites are altered, as in the case depicted in fig. 5(d), we see a shift in the accessibilities of the opposite side of the profile. The leading-term approximation does not capture this shift, and returns a ~ 65% absolute relative error on fig. 5(d). The reason for the discrepancy is that the energies of the outer sites are lowered such that configurations (0, 1) or (1, 0) are similar in energy to (0, 0). Thus the partition function is no longer well approximated by *Z* ≈ *C*_0,0_. We can expand the leading-term approximation to include the next-largest terms in eqs. (2) and (4), *i.e.* those due to the two configurations in which one extra site is unwrapped from either side,

(A.6) $$P_{k}\approx \frac{C_{k,0}+C_{k+1,0}+C_{k,1}}{C_{0,0}+C_{1,0}+C_{0,1}}.$$

Using eq. (A.6) for the left-hand side and a similar expression for the right-hand side recovers all profiles in fig. 5 with < 7% average absolute relative error.
